# 

**Published:** 1852-01

**Authors:** 


### TO READERS AND CORRESPONDENTS.

Communications intended for publication, and all letters relating to the business of the Journal, or containing remittances in money, should be directed to Drs. Harris and Blandy, Baltimore, Md.

### *Books and Pamphlets Received.*

1. *The Teeth and their Preservation in Infancy and Manhood, to Old Age.* By Alfred Canton, M. R. C. S. L., London.

2. The Principles and Practice of Dentistry, simplified and condensed. By R. D. Addington, A. B., D. D. S., Richmond, Va.

3. Ten minutes advice respecting the Human Teeth. By Horace Morton, M. D., Watertown, Mas.

### *Medical and Dental Journals.*

4. *The Western Medico-Chirurgical Journal*, edited by J. F. Sanford, M. D., and Samuel G. Armor, M. D., Professors in Iowa State University, October and November, 1851. *In Exchange.*

5. *The American Journal of the Medical Sciences*, edited by Isaac Hays, M. D. Surgeon to Wills Hospital, &c., October, 1851. *In Exchange.*

6. *The New York Daily Recorder.* Edited by C. C. Allen, M. D., Dentist. September, October and November, 1851. *In Exchange.*

7. *The Dental Register of the West.* Edited by James Taylor, M. D., D. D. S. Published by order of the Mississippi Valley Association of Dental Surgeons. October, 1851. *In Exchange.*

8. *The Dental News Letter.* October, 1851. *In Exchange.*

9. *The London Medical Gazette.* May, August, September and October, 1851. *In Exchange.*

10. *Boston Medical and Surgical Journal.* Edited by J. V. C. Smith, M. D. October, November and December, 1851. *In Exchange.*

### *To Readers and Correspondents.*

11. *The British and Foreign Medico-Chirurgical Review.* October, 1851. *In Exchange.*

12. *The Ohio Medical and Surgical Journal.* Edited by Richard L. Howard, M. D. August and September, 1851. *In Exchange.*

13. *The British American Medical and Physical Journal.* Edited by Archibald Hall, M. D. September, October, November and December, 1851. *In Exchange.*

14. *The New York Journal of Medicine and the Collateral Sciences.* Edited by S. S. Purple, M. D, August, September, November and December, 1851. *In Exchange.*

15. *The Medical Examiner.* Edited by F. G. Smith, M. D., and John Biddle, M. D. October, November and December, 1851. *In Exchange.*

16. *The Southern Medical and Surgical Journal.* Edited by J. P. Garvin, M. D. September, October, November and December. *In Exchange.*

17. *The New York Medical Gazette, and Journal of Health.* Edited by D. M. Reese, M. D., LL. D. October, November and December. *In Exchange.*

18. *The New Jersey Medical Reporter, and Transactions of the New Jersey Medical Society.* Edited by Joseph Parish, M. D. October and November, 1851. *In Exchange.*

19. *The Western Lancet and Hospital Reporter.* Edited by L. M. Lawson, M. D., and George Mendenhall, M. D. September and October, 1851. *In Exchange.*

20. *The North Western Medical and Surgical Journal.* Edited by J. Evans, M. D., and E. G. Meek, A. M., M. D. August, September and October, 1851. *In Exchange.*

21. *The St. Louis Medical and Surgical Journal.* Edited by M. L. Linton, M. D., J. S. Moore, M. D., Wm. McPheeters, M. D., and J. B. Johnson, M. D. Not received since April.

22. *The Western Journal of Medicine and Surgery.* Edited by L. P. Yandell, M. D., and T. S. Bell, M. D. September, October, November and December, 1851. *In Exchange.*

23. *The New Hampshire Journal of Medicine.* Edited by Edward H. Parker, A. M., M. D. September and October, 1851. *In Exchange.*

24. *The Charleston Medical Journal and Review.* Edited by D. J. Cain, M. D., and F. Peyrel Porcher, M. D. August, September and October, 1851. *In Exchange.*

25. *The Buffalo Medical Journal.* Edited by Austin Flint, M. D. September, October, November and December, 1851. *In Exchange.*

### *To Readers and Correspondents.*

26. ***The Provincial Medical and Surgical Journal.*** Edited by W. H. Rankin, M. D., and J. H. Walsh, Esqr. August, September and October, 1851. *In Exchange.*

27. ***The London Lancet***, Journal of British and Foreign, Surgical and Chemical Sciences, Chemistry, Literature and News. Editor Thomas Wakeley, M. D., for London. Sub-Editors, J. H. Bennet, M. D., and T. Wakeley, M. R. C. S. E. October, November and December, 1851. *In Exchange.*

28. ***The Northern Lancet and Gazette of Legal Medicine.*** A monthly Journal of Medical and General Science, Criticism, and Medical Jurisprudence. Edited by Horace Nelson, M. D., and Francis J. D'Avignon, M. D. October, November and December, 1851. *In Exchange.*

29. ***The Dublin Medical Press.*** September, October and November, 1851. *In Exchange.*

---

To Subscribers.—We intend to publish in next number, the list of receipts of payments to second volume New Series, and to all behind we beg to enlist their *early attention.*

---

To Delinquents.—In the July number, we intend to publish a statement of the accounts of all who have failed to pay their subscriptions, after having been *properly solicited a great number of times*, we are compelled to resort to this measure, which we will continue until liquidation.

